# United Kingdom aid cuts: implications for financing health systems

**DOI:** 10.3389/fpubh.2023.1096224

**Published:** 2023-05-10

**Authors:** Kaci Kennedy McDade, Wenhui Mao, Annalisa Prizzon, Ro W. Huang, Osondu Ogbuoji

**Affiliations:** ^1^Center for Policy Impact in Global Health, Duke Global Health Institute, Duke University, Durham, NC, United States; ^2^ODI, London, United Kingdom; ^3^Margolis Center for Health Policy, Duke University, Durham, NC, United States

**Keywords:** official development assistance, donor dependence, donor concentration, health financing, Department for International Development, Foreign Commonwealth and Development Office, foreign aid, health aid

## Abstract

**Background:**

The United Kingdom (UK) used to be the second largest bilateral provider of official development assistance (ODA) for health. However, in 2021 the UK government cut its annual aid budget by 30%. We aim to understand how these cuts might affect financing for health systems in UK aid recipient countries.

**Methods:**

We conducted a retrospective analysis of domestic and external funding for 134 countries that received UK aid for the 2019–2020 budget year. We grouped countries into two cohorts: those that continued to receive aid in 2020–2021 (“budget”) and those that did not (“no budget”). Data was collected from publicly available datasets and we compared UK’s ODA, UK’s health ODA with total ODA, general government expenditures and domestic general government health expenditure to assess the donor dependency and donor concentration of budget and no budget countries.

**Findings:**

Budget countries are more reliant on external aid to finance their governments and health systems than no budget countries, with a handful of exceptions. While the UK does not appear to be a major ODA contributor among most no budget countries, it is in many budget countries. Two no budget countries in particular may be faced with health systems financing challenges given their high ratios of UK health aid to domestic government health expenditures: the Gambia (1.24:1) and Eritrea (0.33:1). Although “safe” for this budget cycle, a number of low-income countries in Sub-Saharan Africa have very high ratios of UK health aid to domestic government health expenditures, including South Sudan (3.15:1), Sierra Leone (0.48:1), and the Democratic Republic of Congo (0.34:1).

**Interpretation:**

The 2021–2022 UK aid cuts could have negative impacts in a few countries highly dependent on UK health aid. Its departure could leave these countries with rather large funding gaps to fill and create a more concentrated donor climate.

## Summary

### What is already known on this topic

Poorly managed foreign aid reductions can have detrimental impacts on a country’s health system. Prior studies have shown that successful donor transitions out of a country require planning and pre-transition investments, the absence of which can lead to discontinuation of programs or deleterious effects on population health.

### What this study adds

Recent changes have been underway in the priorities, size, and allocation of the UK government aid budget. Evidence is needed to identify the potential impacts of the UK’s aid cuts on health system financing among 134 UK aid recipient countries. We provide insights on how much a country will stand to lose, and therefore would need to cover, either by itself or by other donors, to address the budget shortfall following the UK aid cuts in the health sector. This analysis shows how concentrated of a donor environment a country’s health system has, and then puts this concentration in perspective to see how big of a role aid plays within a country’s overall budget and health budget. We identify several countries whose health systems may be at risk given changes to the UK aid funding portfolio.

### How this study might affect research, practice or policy

While the 2021–2022 UK aid cuts may not have catastrophic impact in many countries, it could have negative impacts in a few countries highly dependent on UK health aid. Additionally, its departure narrowed the number of external providers of health aid and created a more concentrated donor climate in many countries. Although 34 countries were spared in this round of budget elimination, these countries may see a reduced budget and should proceed cautiously as discussions of future cuts may be on the horizon. Notably, many low-income countries in Sub-Saharan Africa are particularly reliant on UK funding for financing their health systems. Any sudden policy shift, reduction in funds, or departure could leave these countries with large funding gaps to fill.

## Introduction

In 2019, the United Kingdom (UK) was the third largest provider of foreign aid in low and middle-income countries after the United States and Germany. It is the only G7 country with a commitment to allocate 0.7% of its gross national income (GNI) as Official Development Assistance (ODA) enshrined in law and with the primary objective to contribute to poverty eradication. However, due to recent cuts to its foreign aid budget and shifts in its strategic priorities, the UK reputation as one of the most generous donors and standard setting may soon come to an end. As part of its austerity measures in the aftermath of the Covid-19 pandemic, the UK government abandoned the 0.7% ODA to GNI target down to 0.5% (albeit temporarily and the 0.7% target will be restored only if the fiscal situation improves). As part of this strategy, in 2020 the Department for International Development (DFID), the primary UK agency responsible for managing and disbursing foreign aid, was merged into the Foreign and Commonwealth Office (FCO) (1). The result is a new Department, the Foreign Commonwealth and Development Office (FCDO).

Several months after the merger, the FCDO announced its decision to reduce the number of countries it supports to 34, eliminating the aid budget for 102 countries and territories (2, 3). While these budget cuts are for all types of foreign aid and are not specific to the health sector, they may pose a problem to global health financing given that the UK is a major player in this space; the UK is the second largest bilateral donor for health aid behind the United States (US). But what do these cuts to the bilateral aid programs actually mean for financing health systems in the UK aid recipient countries?

There is a relatively new branch of literature that examines health aid transitions, meaning understanding what happens when health aid is withdrawn from a recipient country in any manner for any reason. A 2022 review identified factors for successful transitions out of health aid as well as key risks that come with these transitions if they are not well managed. In particular, the study found that “*leadership, planning, and pre-transition investments in a country’s financial, technical, and logistical capacity are vital to ensuring smooth transition*” whereas poorly planned transitions “*can result in shortages in financial resources, medical product and supply stock-outs, service disruptions, and shortages in human resources, with resulting implications not only for program continuation, but also for population health*.” (4) In a 2021 study in Ghana, country stakeholders voiced concerns that when donors transition out of the country, they may face “*difficulty filling financial gaps left by donors, the shifting of national priorities away from the health sector, lack of human resources for health, interrupted care for beneficiaries of donor-funded health programs, neglect of vulnerable populations and loss of the accountability mechanisms that are linked with donor financing*.” (5).

The goal of this study is to further the discussion raised in a commentary in the BMJ and put these cuts into perspective (6). We aim to understand how the elimination of budgets might affect health systems’ financing among former UK aid recipient countries. To do so, we first analyze the basic socioeconomic status for “budget” versus “no-budget” countries. While we are most interested in understanding the impact on no-budget countries, it is important to understand the UK’s contribution to the health budgets in budget recipient-countries too; it’s never too early to prepare for a future without foreign aid. Then we explore the potential loss of UK aid, based on the amount of aid a country received in the previous year (2019), and what impact this may have on the country’s budget overall and its health budget. Specifically, we explore three issues: the role of the UK aid out of all external aid sources in a given country, the role of the UK in financing a country, and the role of the UK in financing a country’s health system. This analysis will help us understand how concentrated of a donor environment a country’s health system has, and then puts this concentration in perspective to see what role aid plays within a country’s overall budget and health budget. Additionally, we examine the role other donors play in financing these countries’ health systems to provide comparison to the UK.

## Methods

We conducted a retrospective analysis of domestic and external funding for 134 countries that received UK bilateral aid according to the 2019–2020 budget. We analyzed the ratio of UK official development assistance (ODA) out of all sources of ODA, to the government overall, and specifically to the health sector for budget and no budget countries. We aim to illustrate how much aid may no longer be available for a country to use after aid budget elimination, and therefore, we do not distinguish between the channel of delivery (e.g., via NGOs). For comparison, we also analyzed how much no budget countries rely on other major health donors.

### Approach and variables

The definitions of the indicators used throughout the paper are outlined in Table 1. We used several principles outlined in McDade et al. paper (12). In particular, we similarly looked at issues of donor concentration and donor dependence. There is no agreed up definition in the literature of how these phenomena should be measured and therefore, we proposed adopting the definitions used in our earlier working paper (12). However, we also provided raw data for each country in the analysis to ensure that the reader is able to make a different cutoff determination for what level they may or may not consider to be dependence or concentration (Supplementary file 1).

**Table 1:** Indicators, definitions and data sources.

Indicator	Definition
1. Official development assistance (ODA)	Money that is given or loaned on concessional terms from countries or multilateral institutions to support the welfare or development of lo and middle-income countries. This does not include private donations or other official financial flows that do not meet the concessionality criteria for ODA outlined by the OECD (7).
2. ODA for health	ODA that targets general and basic health, as well as population policies/programs and reproductive health (as defined by the OECD purpose codes 120, 130, 16064) (8). In this study, we focus on bilateral ODA.
3. General government expenditures (GGE)	Total government expenditures across all sectors, including health (9).
4. Domestic general government health expenditure (GGHE-D)	Health expenditures that come from the domestic government (9).
5. UK ODA/Total ODA	UK ODA as a share of total ODA for a given country shows the degree of reliance on UK ODA out of all ODA.
6. ODA/GGE	ODA to GGE shows the degree of a government’s reliance on external aid.
7. UK ODA/GGE	UK ODA to GGE shows the degree of a government’s reliance on UK aid.
8. Health ODA /GGHE-D	Health ODA to GGHE-D shows the reliance of a government’s health system on external health ODA.
9. UK health ODA /GGHE-D	UK health ODA to GGHE-D shows the reliance of a government’s health system on UK health ODA.

The OECD CRS data was used to identify ODA data from external donors (10). We used 2019 disbursement data for our analysis, the latest available data (Indicator 1,2,5,6,7, 8, and 9). The GHED was used to identify domestic government expenditures overall and within the health sector. We used 2018 expenditures data for our analysis, the latest data available (Indicator 3,4,6,8 and 9) (11).

Donor concentration tells us whether a small number of donors make up the majority of ODA, and specifically, if the UK is a major contributor. We used UK ODA/Total ODA to capture donor concentration (Table 1). The primary concern with concentration is that it may create vulnerabilities for a country if one of the few major donors changes its funding level or approach; it could have undue impact on the portfolio of external resources available to a country.

Donor dependence on the other hand tells us whether or not a country relies heavily on external resources in comparison to what the domestic government spends. In the previously mentioned paper by McDade et al., dependency was defined as a ratio of external aid to domestic expenditures of 0.25:1. In other words, they believe a country would be considered dependent if donors financed 25 cents or more for every dollar the domestic government spends. The key concern with dependency is whether or not a country would find it difficult to fill such a gap in the event of a donor exit. In this paper, however, we are not making a judgment on whether or not these countries are “dependent” on UK aid. We simply used this principle to help portray how much of a gap a country may need to fill given aid cuts. The indicators used to assess reliance on external resources are listed in Table 1 (Indicators 6–9).

We conducted summary statistics for each of these indicators. Specifically, we compared the median (with 25th and 75th percentiles) between no budget and budget countries and visualize the distribution of individual countries by region (Table 1). We opted to use the median with 25th and 75th percentiles based on the skewed distribution of data.

### Data sources

Our primary data sources were the World Bank, Global Health Expenditure Database (GHED), the United Nations (UN) Department of Economic and Social Affairs, and the Organisation for Economic Co-operation and Development’s (OECD) Creditor Reporting System (CRS; Table 1). World Bank 2022 fiscal year data was used for country income-level, region, size (i.e., small states, or those with less than 1.5 million population size), and level of fragility (13–15). We included these country characteristics to add context to the type of countries that do/do not receive budgets from the UK in the 2020–2021 aid year since these characteristics indicate varying types of development challenges. For example, small states, according to the World Bank, “are particularly vulnerable to exogenous shocks” (15). We recognize that fragility is defined in various ways depending on the source. Given our use of the World Bank classifications for other country characteristics, we used their classification of fragility and conflict for (a) consistency and (b) because all underlying indicators are publicly available. All financial data are reported in 2019 US dollars.

We used Devex’s list of budget and no budget countries/territories (2). Two territories did not have any data available in databases and therefore were excluded entirely: Montserrat and St. Helena. Seven countries/territories had data for overarching characteristics, such as income level and fragility, but limited data across other various indicators. These countries/territories are: Democratic Republic of Korea, Kosovo, Libya, Somalia, Syria, West Bank & Gaza Strip, and Yemen. We have therefore only included them in the characteristics section and dropped them from the subsequent analyses. We have indicated the sample size for each indicator in Table 1.

## Results

### Characteristics of budget and no budget countries

In total, we analyzed the characteristics of 134 countries: 34 countries that continued to have a budget for the 2020–2021 aid year and 100 countries that did not have a budget for the 2020–2021 aid year. We assessed their income level, region, size, and fragility status, according to the World Bank classification.

**Figure 1:**
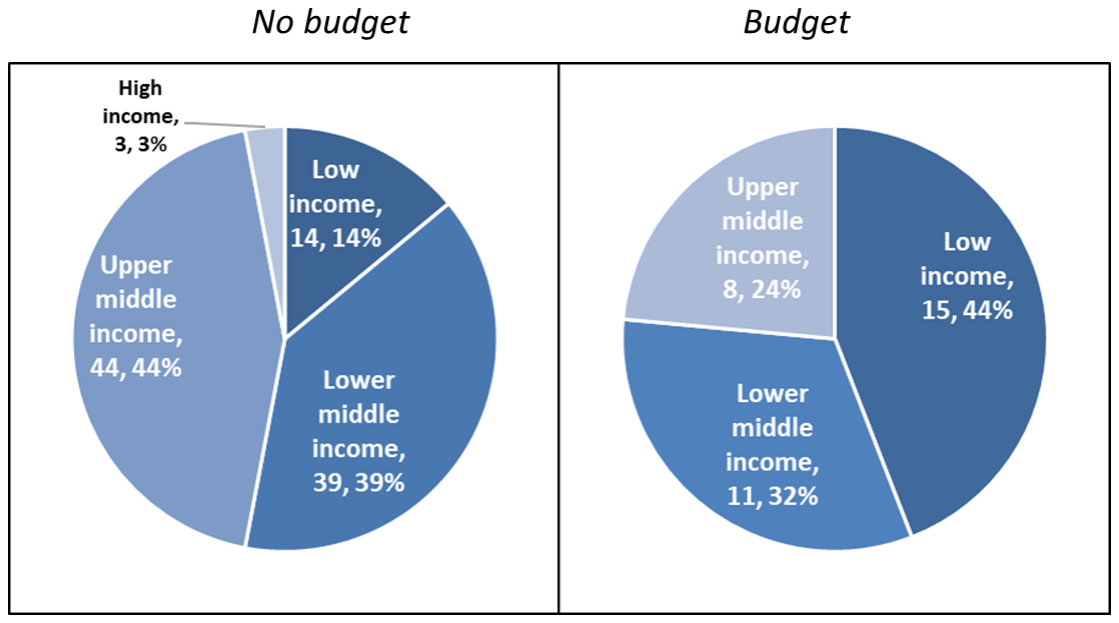
Income level of no budget and budget countries.

More than half of all no budget countries are low or lower-middle income (*n* = 53, 53%) while 44% are upper-middle income (*n* = 44), and 3% are high-income (*n* = 3; Figure 1). The median GDP *per capita* of no budget countries is US$3,861 (Table 2). More than a quarter of countries are in sub-Saharan Africa (*n* = 27, 27%) followed by Latin America and the Caribbean (*n* = 23, 23%). Nearly a quarter of countries are considered fragile or conflict-affected (*n* = 24, 24%) while a third are small states (*n* = 34, 34%).

**Table 2:** Summary statistics of no budget and budget countries.

Indicator	No budget (*n* = 100)	Budget (*n* = 34)
GDP *per capita* (USD)	3,861 [1858, 6,289]	1,339 [661, 3,891]
UK ODA/Total ODA (%)	1% [0, 2%]	7% [4, 9%]
UK health ODA/Health ODA (%) *	0 [0,0]	7% [2, 12%]
ODA/GGE *	0.09 [0.03, 0.29]	0.15 [0.01, 0.45]
UK ODA/GGE *	0 [0,0]	0.01 [0,0.03]
Health ODA/GGHE-D *	0.06 [0.01, 0.39]	0.41 [0.02, 2.06]
UK health ODA/GGHE-D *	0 [0,0]	0.02 [0,0.08]

Data reported: median [25th percentile, 75th percentile], 0 represents the value is less than 0.01.

Seven countries/territories had incomplete data for variables marked with “*”, and sample size for those variables were 96 and 31 for no budget and safe group, respectively.

Three-quarters of all budget countries are low or lower-middle income countries (*n* = 26, 76%) whereas 24% are upper-middle countries (*n* = 8; Figure 1). The median GDP *per capita* of budget countries is US$ 1,339 (Table 2). More than half are in sub-Saharan Africa (*n* = 19, 56%) followed by South and East Asia (*n* = 8, 24%). Over a third (*n* = 13, 38%) are considered fragile-or conflict affected countries. None are considered small states.

Overall, budget countries are more reliant on external aid to finance their governments and health systems (Table 2). Budget countries have higher median ratios of ODA to GGE and Health ODA to GGHE-D than no budget countries. While the UK’s role in financing both is minimal, the UK is generally a more prominent donor among budget countries (Table 2). Although both groups of countries exhibit “need” based on their income-level and fragility status, a larger share of budget countries are lower-income and fragile.

### The role of UK ODA within the broader ODA landscape

Since the UK aid cuts are not specific to the health sector, we assess the role the UK contributes to a country’s total ODA across all sectors in addition to a country’s health-specific ODA. In general, we find that the UK contributes a larger share of total ODA and health ODA in budget countries than in no budget countries, with some exceptions.

**Figure 2:**
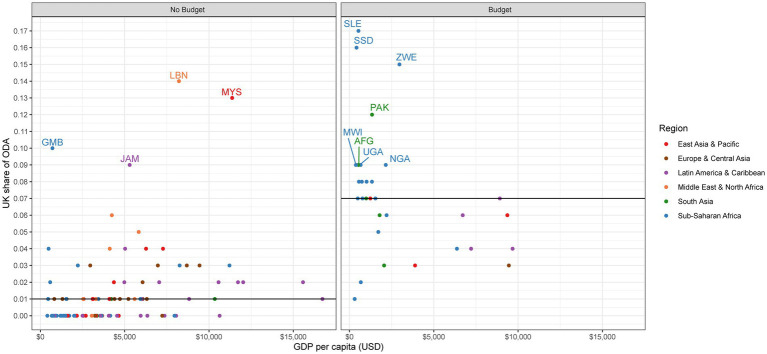
UK share of ODA.

**Figure 3:**
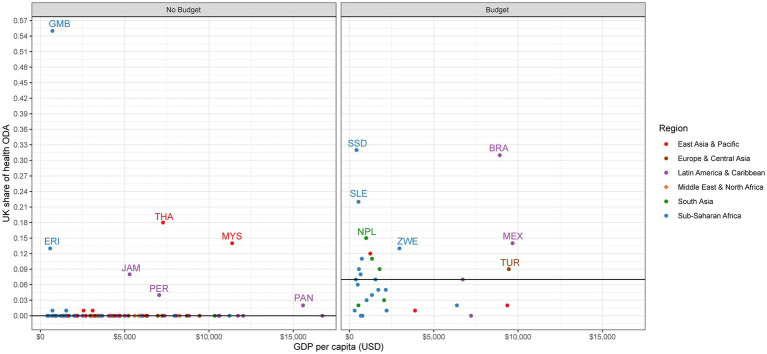
UK share of health ODA.

For no budget countries, the median share of UK ODA out of total ODA is 1% (Table 2). Although the UK contributes a small share across most no budget countries, the UK does make up a larger share of ODA in some. Specifically, UK ODA contributes the largest share in Lebanon (14%), Malaysia (13%), the Gambia (10%), and Jamaica (9%; Figure 2). For health ODA specifically, the median share of UK health ODA out of all health ODA is 0% (Table 2). However, there are several exceptions where the UK is a large player, such as the Gambia (55%), Thailand (18%), Libya (14%), Malaysia (14%), Eritrea (13%), and Jamaica (8%; Figure 3).

United Kingdom ODA plays a more prominent role in budget countries for both total ODA and health-specific ODA. The median share for both UK ODA out of total ODA and UK health ODA out of health ODA is 7% (Table 2). In terms of total ODA, the UK is one of the top donors in several countries, including Sierra Leone (17%), South Sudan (16%), Zimbabwe (15%), Somalia (13%), and Pakistan (12%; Figure 2). We see even higher shares of UK contributions within health ODA, with the highest shares found in South Sudan (32%), Brazil (31%), Sierra Leone (22%), Somalia (20%), and Nepal (15%; Figure 3).

Assessing the role UK ODA plays across all sectors, and within the health sector, shows us how big of a player the UK is compared with other external donors. The UK is not a large player in the vast majority of countries that will not receive a budget for the 2021–2022 year and is a larger player in many of the countries that will still see continued aid programs. However, this indicator does point to several no budget countries where UK aid does make up a considerable portion of total ODA, and therefore could feel the impacts of UK exit more acutely.

### The role of ODA in comparison to domestic spending

To put into perspective how much external aid plays a role in financing a country’s government, and specifically to what extent the UK contributes to the pool of external resources, we assess the ratio of ODA to GGE and UK ODA to GGE. We find that ODA plays a considerable role in financing many countries across both groups. However, the extent to which the UK contributes is limited in most no budget and budget countries, with a few notable exceptions.

**Figure 4:**
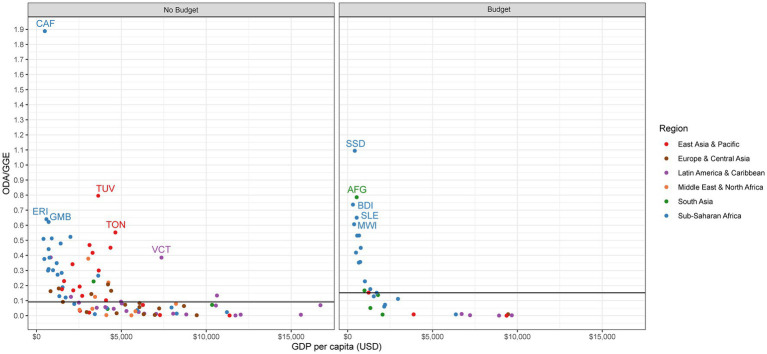
ODA to GGE.

**Figure 5:**
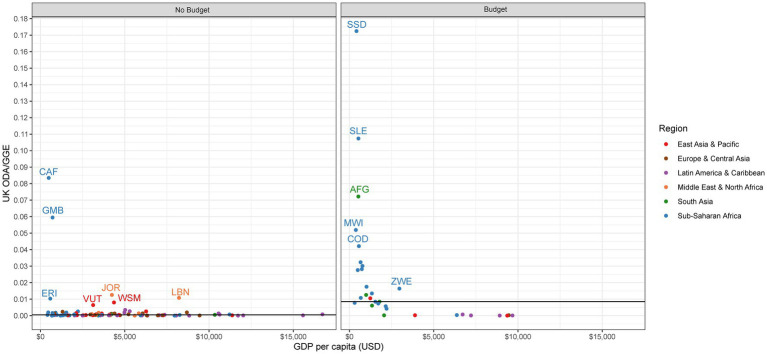
UK ODA to GGE.

For no budget countries, the median ratio of ODA to GGE is 0.09:1, meaning that for every nine cents donors spend, the government spends a dollar (Table 2). Twenty-five no budget countries have a ratio of ODA to GGE of 0.25:1 or greater, meaning that for these countries, external donors spend 25 cents or more for every dollar the government spends (Figure 4). In all but two countries (the Central African Republic and the Gambia), the ratio of UK ODA to GGE is nearly zero, meaning that the UK is not a major contributor to ODA (Figure 5).

The median ratio of ODA to GGE among budget countries is 0.15:1, meaning that for every 15 cents donors spend, the government spends a dollar (Table 2). A third of budget countries have ODA to GGE ratios of 0.25:1 or greater (*n* = 11), most of which are located in Sub-Saharan Africa and are low-income (Figure 4). This threshold of 0.25:1 was used in McDade et al. to signal donor dependency (12). The reliance on the UK as a source of ODA is greater among budget countries than in no budget countries, albeit the UK is overall still a relatively small contributor to ODA in these countries. The UK ODA to GGE ratio is highest in South Sudan (0.17:1), Sierra Leone (0.11:1), and Afghanistan (0.7:1; Figure 5).

A number of both no budget and budget countries receive considerable amounts of ODA compared to their own government’s general expenditures. Although the UK does not seem to be a major contributor to high ratios of ODA to GGE, there are several countries, both no budget and budget, where it plays a considerable role, including the Central African Republic, the Gambia, South Sudan, Sierra Leone, and Afghanistan. These countries in particular will have the most external funds relative to their own expenditures that will need to be found from elsewhere in the event of a donor exit.

### The role of health ODA in comparison to domestic health sector expenditures

Looking specifically within the health sector, we analyze the ratio of health ODA to domestic government expenditures on health (GGHE-D). We focus on GGHE-D rather than all domestic financing sources since the government would be the main party accountable for financing the health system gaps in the event of a donor exit. However, we do recognize such funding gaps could in reality be filled by other sources, such as out of pocket payments by consumers.

We find a strong divergence between no budget countries and budget countries in terms of external health aid to domestic health expenditures. Although a number of no budget countries do have high ratios of health ODA to GGHE-D, the median ratio across budget countries is considerably higher (0.41:1) than in no budget countries (0.06:1; Table 2). While the UK does not appear to be a top contributor to health sector dependency on aid among zero-budget countries, it does seem to be in many budget countries.

**Figure 6:**
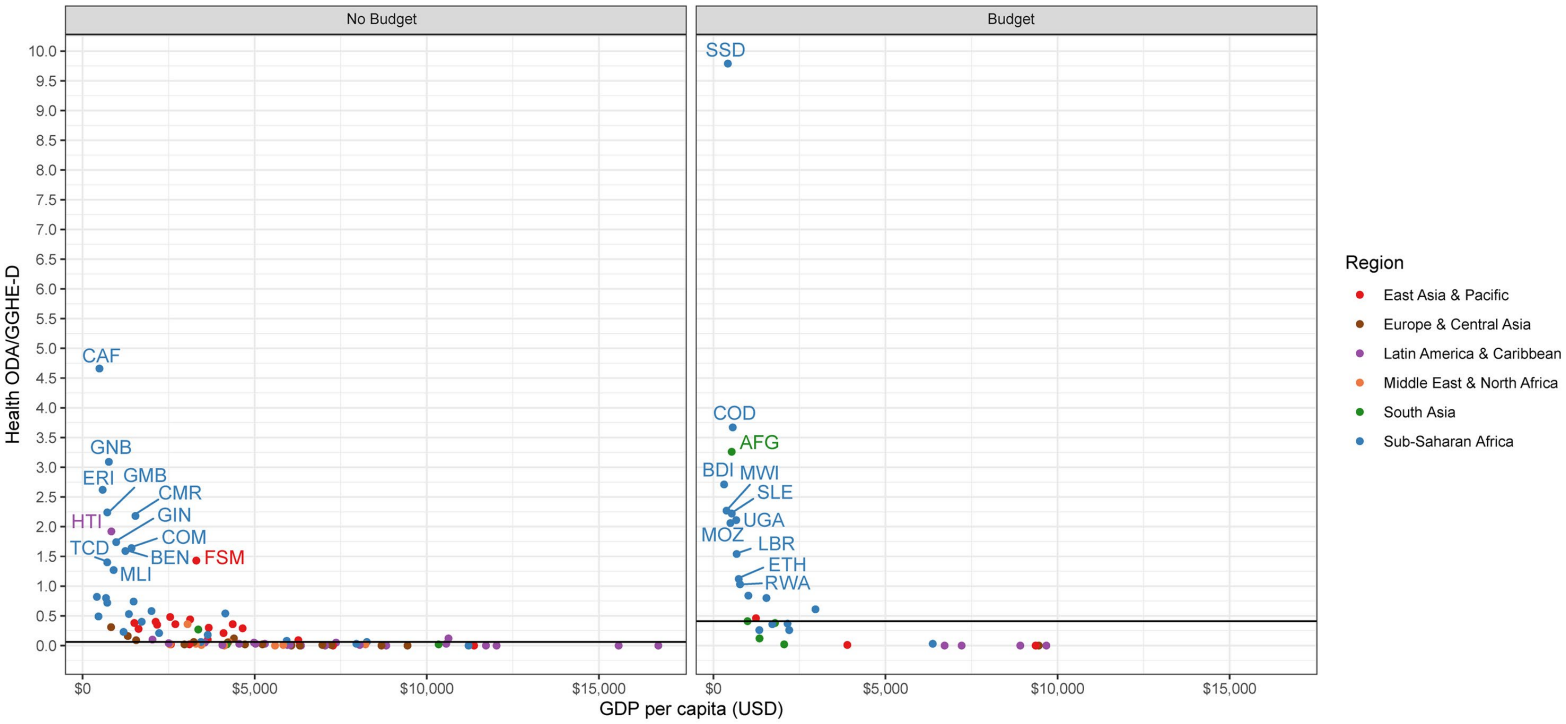
Health ODA to GGHE-D.

No budget countries have a median health ODA to GGHE-D ratio of 0.06:1 (Table 2). One-third of no budget countries have a ratio of health ODA to GGHE-D greater than 0.25:1, an indicator that has been previously used to signal donor dependency in the health sector (Figure 6) (12). This means that in a third of the no budget countries, health ODA is a major source of financing for the health system: for every $1 the government spends on health, external donors spend more than 25 cents. In 12 countries, this ratio exceeds 1:1, meaning health aid contributes more to the health system than the domestic government: Central African Republic (4.7:1), Guinea-Bissau (3.1:1), Eritrea (2.6:1), Gambia (2.2:1), Cameroon (2.2:1), Haiti (1.9:1), Guinea (1.7:1), Comoros (1.6:1), Benin (1.6:1), Micronesia (1.4:1), Chad (1.4:1), and Mali (1.3:1). If we examine the ratio of UK health aid to GGHE-D, we can see how the UK spending on health compares to the domestic government. Among no budget countries, the median ratio of UK health ODA to GGHE-D is 0.0:1 (Table 2). Only two no budget countries have a sizeable ratio: the Gambia (1.24:1) and Eritrea (0.33:1; Figure 7).

**Figure 7:**
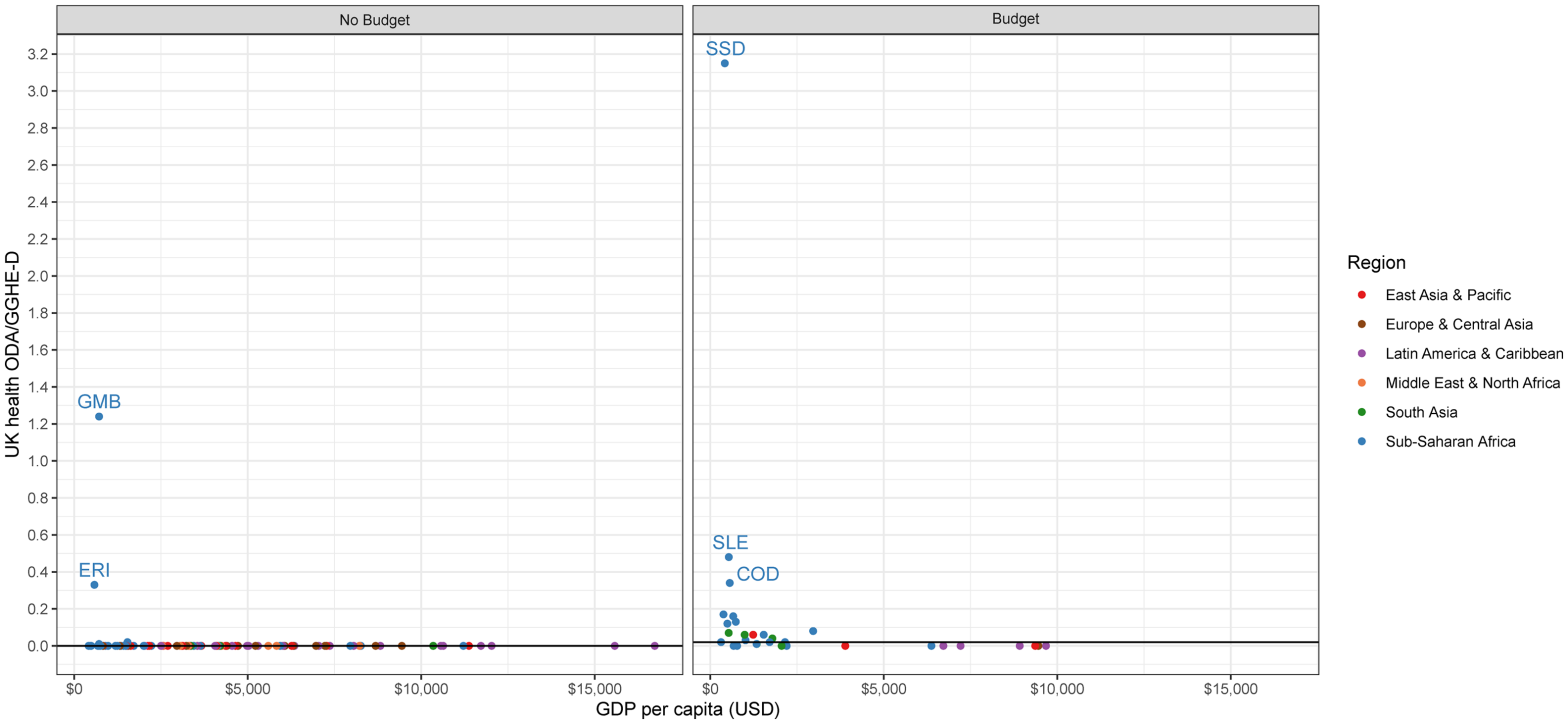
UK health ODA to GGHE-D.

For budget countries, we see aid play a much stronger role in financing health. Among budget countries, the median health ODA to GGHE-D ratio is 0.41:1, meaning external donors contribute $0.41 for every $1 the domestic government spends (Table 2). 62% of budget countries (n = 21) have a dependency ratio of 0.25:1 or greater, signaling potential dependency on external aid to finance their health systems (Figure 6). The role of UK health aid varies among these budget countries, however (Figure 7). While the median ratio of UK health ODA to GGHE-D is 0.02:1, several low-income countries in Sub-Saharan Africa have very high ratios, such as South Sudan (3.15:1), Sierra Leone (0.48:1), and the Democratic Republic of Congo (0.34:1), suggesting that donors match every US$1 spent by the host country by US$ 3.15, US$ 0.48, and US$0.34, respectively. While any change in UK spending in these three countries would likely be strongly felt, it is important to note that these countries are also all dependent on US support, in some cases even more so than they are on the UK: South Sudan (0.72:1), Sierra Leone (0.51:1), and the Democratic Republic of Congo (0.52:1). If the UK were to change its policy in these countries, these countries would then have a more concentrated donor environment and be even more reliant on other existing donors, such as the US. These countries are also dependent on financing from Canada and Germany, although to a lesser extent.

## Discussion

The UK’s budget cut may not be as catastrophic for many health systems as expected. However, some no budget countries may be impacted by the UK departure more than others, particularly the Gambia and Eritrea. While the absolute value of health ODA from the UK may be small in some countries, like Thailand, Libya, or Malaysia, they have a concentrated donor environment and may feel the effects of the UK’s absence more than other countries. Fewer donors means less bargaining power for countries. Many no budget countries are reliant on other donor resources, particularly resources from the US. UK exit, narrows the number of external players willing to support the health system and could lead to even more donor concentration.

Overall, the UK is a larger player in budget countries than no budget countries. Several of these budget countries, particularly low-income countries in Sub-Saharan Africa, show high ratios of reliance on UK health aid. Despite being “safe” for this upcoming year, budget countries should not be relieved since they could face cuts in the near future. Some insights signal that a third round of cuts may be in store in spring 2022, potentially causing the UK to become a significantly smaller bilateral aid provider (16). More should be done to enable self-reliance in budget countries and ensure when a transition out of UK aid inevitably happens, it can be done in a sustainable manner.

While the majority of the budget countries demonstrate “need” in terms of their income-level, many are upper-middle income countries, particularly those with strategic interest to the UK as highlighted in the 2022 UK government’s strategy for international development, notably the Indo Pacific region. This prioritization of some wealthier countries demonstrates FCDOs departure from many of DFID’s norms. For example, several of the upper-middle income countries that FCDO will continue to fund are those which DFID had previously transitioned or exited (India, Indonesia, South Africa) (17). Additionally, while nearly half of the no budget countries are wealthier, over half are still among the world’s neediest. Other analyses have shown that UK aid cuts have disproportionately impacted the poorest and most fragile countries (18). FCDO has an opportunity to better target its future resources towards the world’s neediest countries.

Another concerning change is that DFID was bound to support poverty reduction, while other aid disbursing agencies, such as the FCO, were not required to do so (19). This merger may compromise DFID priorities (i.e., issues facing the world’s poorest and neediest) in favor of FCO priorities that may serve more geopolitical strategic interests (20). Other concerns of the merger included compromising the reputation of transparency and evaluation DFID has acquired over the years; according to Publish What You Fund in 2020, DFID was considered one of the most transparent bilateral funders while the FCO was one of the least (21, 22). In 2022 the FCDO ranked 16^th^, compared to DFID 9^th^ in 2020 (21).

Our study has several limitations. First, we focus solely on financing but there certainly are other considerations worth investigating that span beyond financial loss, particularly as it relates to health outcomes. Even if funding amounts are small, the UK could be providing critical technical assistance or monitoring, the loss of which might undermine future health outcomes. Second, we do not propose ways to close this financing gap but rather illustrate the size of the gap countries may have to close with domestic or external resources. Third, we believe we have used the best data sources available for this analysis. However, our approach and selections are not without limitations. We recognize that OECD reports disbursements while GHED reports expenditures and that these figures are not interchangeable. Ideally, we would have used GHED data on external sources of funding (EXT) since this indicator is more encompassing than ODA, however, EXT data is often incomplete or missing and therefore was not used. Given limited data availability for the new FCDO on the UK’s DevTracker, and the agency’s recent establishment, the OECD was our best resource. We recognize that using overall UK ODA instead of agency specific ODA may overestimate the amount of ODA from the FCDO. However, as the primary provider of UK ODA, we believe this an appropriate proxy.

## Conclusion

The 2021–2022 UK aid cuts could have negative impacts in a few countries highly dependent on UK health aid. Its departure narrowed the number of external providers of health aid and created a more concentrated donor climate in many countries. Additionally, 34 countries were spared in this round of budget elimination yet many still saw reductions in their UK aid budgets. Many of these 34 countries are low-income countries located in Sub-Saharan Africa, and are particularly reliant on UK funding for financing their health systems. Any sudden policy shift, reduction in funds, or departure could leave these countries with rather large funding gaps to fill.

## Transparency statement

WM affirms that the manuscript is an honest, accurate, and transparent account of the study being reported; that no important aspects of the study have been omitted; and that any discrepancies from the study as originally planned (and, if relevant, registered) have been explained.

## Data availability statement

Publicly available datasets were analyzed in this study. This data can be found here: https://www.oecd.org/tax/automatic-exchange/common-reporting-standard/ and https://apps.who.int/nha/database.

## Author contributions

KM, WM, and OO conceptualized the study. KM, WM, AP, and RH conducted the analysis with input from OO. KM wrote the first draft of the study. All authors contributed to the article and approved the submitted version.

## Funding

This paper was part of the project “Driving health progress during disease, demographic, domestic finance and donor transitions (the “4Ds”): policy analysis and engagement with six transitioning countries” funded by Bill and Melinda Gates Foundation (OPP1199624).

## Conflict of interest

The authors declare that the research was conducted in the absence of any commercial or financial relationships that could be construed as a potential conflict of interest.

## Publisher’s note

All claims expressed in this article are solely those of the authors and do not necessarily represent those of their affiliated organizations, or those of the publisher, the editors and the reviewers. Any product that may be evaluated in this article, or claim that may be made by its manufacturer, is not guaranteed or endorsed by the publisher.

## Abbreviations

CRS: Creditor Reporting System
DFID: Department for International Development
EXT: external sources of funding
FCDO: Foreign Commonwealth and Development Office
FCO: Foreign and Commonwealth Office
GGE: General government expenditures
GGHE-D: Domestic general government health expenditure
GHED: Global Health Expenditure Database
GNI: Gross national income
ICAI: Independent Commission for Aid Impact
LMICs: low and middle-income countries
ODA: official development assistance
OECD: Organization for Economic Development and Cooperation
UK: United Kingdom
UN: United Nations
US: United States
